# Growth Modeling of the Giant Electric Ray *Narcine entemedor* in the Southern Gulf of California: Analyzing the Uncertainty of Three Data Sets

**DOI:** 10.3390/ani12010019

**Published:** 2021-12-23

**Authors:** Pablo Mora-Zamacona, Felipe N. Melo-Barrera, Víctor H. Cruz-Escalona, Andrés F. Navia, Enrique Morales-Bojórquez, Xchel A. Pérez-Palafox, Paola A. Mejía-Falla

**Affiliations:** 1 Centro Interdisciplinario de Ciencias Marinas, Instituto Politécnico Nacional, Baja California Sur, La Paz 23096, Mexico; pablomoraz90@gmail.com (P.M.-Z.); fmelo@ipn.mx (F.N.M.-B.); xapp39@gmail.com (X.A.P.-P.); 2 Fundación Colombiana para la Investigación Y Conservación de Tiburones Y Rayas, SQUALUS, Calle 10A No 72-35, Apto. 310 E, Cali 760001, Colombia; anavia@squalus.org (A.F.N.); pmejia@squalus.org (P.A.M.-F.); 3 Centro de Investigaciones Biológicas del Noroeste, Av. Instituto Politécnico Nacional 195, Col. Playa Palo de Santa Rita Sur, La Paz 23096, Mexico; emorales@cibnor.mx

**Keywords:** elasmobranchs, individual growth modelling, artisanal fisheries, multimodel inference, back-calculation

## Abstract

**Simple Summary:**

The study of age and growth patterns in skates and rays can be conducted by analyzing mineral deposition patterns inside the vertebrae as biological features may influence age estimation. For the giant electric ray (*Narcine entemedor*), age was estimated by analyzing the vertebrae and an annual deposition pattern was found. After considering additional biological features such as birth date and date of capture, a more precise description of growth pattern was made. We concluded that this species is a moderate body size elasmobranch with moderate longevity and fast growth. Our results provide useful information for the future management of this exploited species.

**Abstract:**

The age and growth rate of the giant electric ray, *Narcine entemedor*, was estimated using growth bands deposited in the vertebral centra of 245 specimens. Differences in size and age distribution were found between the sexes, a pattern that suggests the annual deposition of band pairs, possibly occurring in April. Multimodel inference and back-calculation were performed to three age data sets of females considering their reproductive cycle and time of capture, among which the von Bertalanffy growth function was found to be the most appropriate (*L*_∞_ = 81.87 cm TL, *k* = 0.17 year^−1^). Our research supports the idea that age can be determined via biological features such as birth date and growth band periodicity. We concluded that *N. entemedor* is of a moderate body size, moderate longevity and is a fast-growing elasmobranch species.

## 1. Introduction

Traditionally, it has been recognized that elasmobranchs, due to their biological characteristics (late maturation, low fecundity, slow growth), are especially vulnerable to overfishing [1,2,3,4]. Nevertheless, evidence has proven that life-history traits of this group may vary between K and r strategies, allowing some species to respond differently to fishing pressure [5,6,7]. In this sense, it is important to understand the life-history parameters of the species, particularly those that are related to the degree of vulnerability and risk to fishing pressure, providing basic information for demographic models [8,9,10]. Among the most important parameters for this purpose are the ones related to the age and growth of the species [11].

The number of age and growth studies in elasmobranchs has increased significantly in recent years [12]; however, the techniques and structures used for these purposes have remained constant, based mainly on the identification and count of opaque and translucent banding patterns present in hard anatomical structures such as vertebral centra, denticles and dorsal spines [13,14].

Many studies on the age and growth of elasmobranchs have been encouraged by the increasing exploitation of this group, which has been documented by numerous researchers, reporting the important effects of fishing mortality on batoid populations [15,16,17,18]. In Mexican elasmobranch fisheries, the main species of batoids captured are benthic, such as *Pseudobatos productus*, *Zapteryx exasperate* and *Hypanus dipterurus* [19,20,21,22], although some pelagic species such as *Pteroplatytrygon violacea* and *Myliobatis californica* have been found frequently in the catch [22,23]. In all these study cases, *Narcine entemedor* (Jordan and Starks, 1895) have been reported with low abundances; nevertheless, Villavicencio-Garayzar [24] and Márquez-Farías [25] reported that they are commonly captured in artisanal fisheries of Mexico, especially during spring and summer. In Bahía de La Paz, an artisanal fishery on batoids captures around 14 species, among which *N. entemedor* ranks third in captures [26].

The giant electric ray *N. entemedor* is an endemic batoid of the eastern tropical Pacific Ocean distributed from southern Baja California and the Gulf of California to Peru. While the diet and feeding ecology [27] and reproductive biology [24,28] of the giant electric ray have been investigated, there is little information on the age and growth of this species. Moreover, information on the species is limited to the Pacific coast of Mexico, while life-history traits could vary in Central and South America. The objective of this study was to estimate age and growth parameters for *N. entemedor* in the southern Gulf of California using a multimodel inference approach, and to consider the influence of biological features in the estimates. We conclude that using data such as birth date may result in a more precise description of individual growth.

## 2. Materials and Methods

### 2.1. Collection of Samples

The specimens were captured in collaboration with a fisherman who has a commercial fishing permit under Mexican fishing regulations and laws (CONAPESCA-103053993316-1); therefore, the data in this study are fishery-dependent. Monthly samplings were made from October 2013 through December 2015 in the south of Bahía de La Paz, located in the southern portion of the Gulf of California (24°25′ N, 110°18′ W). The organisms were captured using monofilament gill nets (200–300 m long, 1.5 m high, 20–25 cm stretch mesh) set in the afternoon at depths between 10 and 30 m over sandy bottoms and recovered the next morning. Individuals were measured for total length (TL in cm), and the sex was determined by the presence of copulatory organs in males (claspers; [28]). Vertebrae were collected from the abdominal region of each specimen and placed in a freezer.

### 2.2. Vertebral Preparation

Vertebrae were thawed, excess tissue manually removed and individual centra were separated using a scalpel. Cleaned vertebrae centra were dried at room temperature. A qualitative analysis to test the effectiveness of diverse treatments to enhance the visibility of growth bands was evaluated. Three cutting thicknesses (0.3, 0.4 and 0.5 mm) and two dyes, Alizarin Red S (0.01 g/250 mL water) and Bismarck Brown Y (0.01 g/250 mL alcohol 95%), were tested to enhance the visibility of the centra growth bands. Staining was performed at different times of exposure, from 1 min to saturation. However, none of the dyes tested presented an improvement in the clarity of growth bands; thus, vertebrae were instead prepared as follows. Centra were fixed on wooden structures using a cyanoacrylate-based adhesive. Sagittal sections of 0.4 mm thick were made using double saws fitted with diamond-impregnated blades, ensuring that the focus of the centrum was included. Subsequently, sections were cleaned with scalpel and water and then dried at room temperature.

### 2.3. Reading of Growth Bands

Thin sections were observed under a stereo microscope (Olympus SZX9) and digitized using a video camera (Sony CCD-IRIS-RGB). The sections were illuminated using reflected light on a dark background and submerged in a thin layer of water to improve the observation. The birth band was defined as the angle change on the centrum side [29]. Additionally, a centrum of a 12.4 cm TL near-term embryo [28] was polished and compared with a thin section of an adult. The size of the embryo’s centra and the birth band coincided; furthermore, no pre-birth bands were observed. The radius of each vertebra was measured on the corpus calcareum long a straight line through the focus of each vertebra with SigmaScan Pro 5.0.0 Software (Systat Software, Palo Alto, CA, US). The vertebral radius (VR) was plotted against TL and tested for a linear relationship to determine if these vertebrae provided a suitable structure for age determination and for back-calculated estimation of length at previous ages. The influence of sex in the VR-TL relationship was evaluated with a test of slopes and elevations [30].

**Figure 1:**
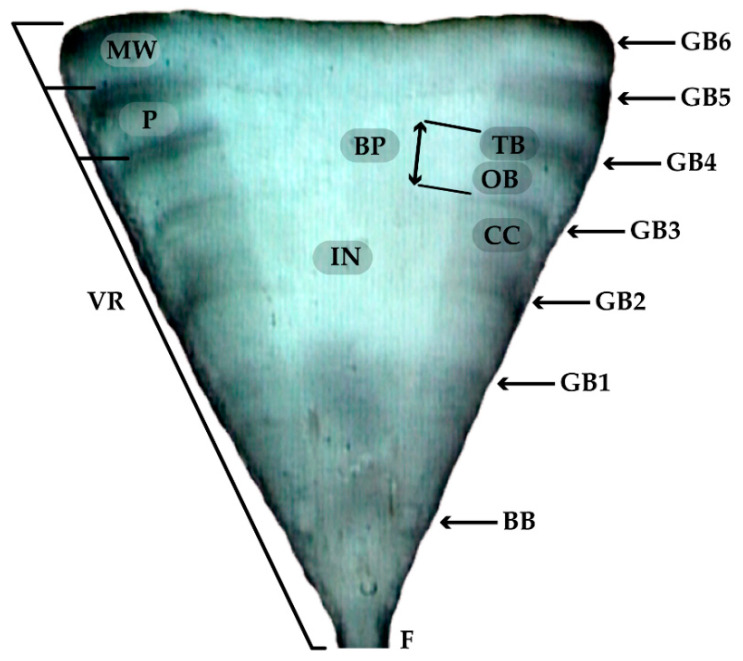
Sagittal section of a centrum of a six-year-old *Narcine entemedor* specimen, showing MW, marginal width; P, previous band pair width; VR, the vertebral radius; F, focus; CC, corpus calcareum; IN, intermedialia zone; BP, band pair; OB, opaque band; TB, translucent band; BB, birth band; GB, growth bands.

A training exercise counting the bands of a subsample (n = 50) was performed by two readers to refine the identification and growth band counts criteria. The following criteria were established: (1) identification of the presence of pairs of growth bands (one translucent and one opaque; [31]), (2) identification of a birth band through a change in the angle of the corpus calcareum in the place closest to the focus of the vertebra [29] and (3) counting of translucent bands (Figure 1). The two readers (reader 1 was most experienced) then did a simultaneous and independent band count without knowing the sex or size of the specimens. Readings of bands were performed on the corpus calcareum due to the poor visibility in the intermedialia zone. This procedure was repeated twice. Any vertebra yielding an age estimate that differed between counts was re-examined by both readers jointly; if no consensus was reached, the sample was discarded.

### 2.4. Precision and Accuracy

According to Campana [31], aging errors can be expressed as follows: (a) discrepancies in the reproducibility of repeated measurements on a given structure (precision), and (b) differences between the closeness of the age estimate to the true value (accuracy). Thus, count reproducibility, as indicated by reader variability, was determined by calculating the percent of agreement (PA) by ±1 mark [32], average percent error (APE; [33]) and coefficient of variation, which is an alternative precision analysis that uses the standard deviation rather than the absolute deviation (CV; [34]). Each method was applied to the total of the sample. Additionally, to assess whether there are systematic differences between the readings made by the readers, age-bias plots of band counts [35] and Bowker’s test of symmetry were performed [36].

### 2.5. Periodicity of Band Formation

The periodicity of band pair formation was evaluated using two methods. The “centrum edge analysis” considers whether the last deposited band was translucent or opaque and relates it to the month of capture [37]. The “marginal increment analysis” was undertaken by measuring the distance from the last band to the edge of the centrum (marginal width) as a proportion of the distance between the last and the penultimate band pair (previous band pair width; [38]).

(1) $$MIR=\frac{MW}{P}=\frac{VR-R_{n}}{R_{n}-R_{n-1}}$$

where *MIR* = the marginal increment ratio, *MW* = marginal width, *P* = previous band pair width, *VR* = the vertebral radius, *R_n_* = the distance from the focus to the last complete growth mark band and *R_n_*_−1_ = the distance from the centrum origin to the penultimate complete growth mark(Figure 1). Those distances were measured using SigmaScan Pro 5.0.0 Software (SPSS Inc., Chicago, IL, USA). Individuals that presented only one translucent band (referred to as the birth band) were not considered for MIR analysis. Mean MIRs were plotted against months to examine trends in band formation. A Kruskal–Wallis test was used to examine for differences among months, followed by a nonparametric multiple comparison test to find the months among these differences were presented [30]. In addition, a Kolmogorov–Smirnov distribution test was used to examine for differences in age structures between sexes [39].

### 2.6. Age Adjustment

For the individual age estimation, three data sets were analyzed, one using the growth band counts information (unadjusted) and two more performing an adjustment to age, considering reproductive cycle (Adjustment 1) and time of capture (Adjustment 2). For the unadjusted analysis, it was considered that the birth band formed shortly after parturition, irrespective of reproductive seasonality. Adjustment 1 was adjusted to the period between birth and first band formation. In the study area, *N. entemedor* has two birth peaks per year, a major one during August and a minor one during January [28]; the month of band formation is April (see Section 3). We considered the birth month to be August; thus, the time between birth and formation of the first band was assumed to be seven months (0.58 years). Adjustment 2, the second age adjustment integrated the capture date; therefore, age was adjusted with the time between the month of band formation and capture month.

### 2.7. Back-Calculation

Due to the small sample size of juvenile giant electric rays, back-calculated estimates of length at previous ages were calculated for each of the three data sets. Back-calculated lengths were calculated using the proportion-based back-calculation equation proposed by Francis [40], modified from Hile [41]. The equation used to back-calculate the lengths at presumed ages was:

(2) $$L_{i}=-\left(\frac{a}{b}\right)+\left(L_{c}+\frac{a}{b}\right)\left(\frac{VR_{i}}{VR_{c}}\right)$$

where *L_i_* is the TL at time *i*, a and b are parameters obtained from the linear relation between total length and vertebral radius, *L_c_* is the TL at capture; *VR_c_* is the *VR* at capture and *VR_i_* is the *VR* at age *i*.

### 2.8. Growth Estimation

A multimodel inference approach was used to determine the most appropriate candidate growth model [42]. The candidate set of models consisted of the traditional 3-parameter von Bertalanffy growth model (VBG-3; [43]); a 2-parameter modified form of the VBG forced through the length-at-birth (*L*_0_) (VBG–2; [44]), where *L*_0_ was estimated using the largest near-term embryo (i.e., 14.5 cm TL) reported by Burgos-Vázquez et al. [28]; the 3-parameter Gompertz growth model (GG-3) and the logistic model with three parameters (LG-3; [45]).

The four growth models were fitted to a combination of back-calculated lengths and sample data for the three data sets previously described (unadjusted and adjusted ages), and the resulting parameters were estimated and compared. The parameters in the candidate growth models were estimated when the negative log-likelihood was maximized with a nonlinear fit using the generalized reduced gradient method, assuming a multiplicative error in the residuals [46,47]. The objective function is expressed as follows:

(3) $$-logL\left(\theta_{i}|data\right)=\underset{n}{\sum }\left[-\frac{1}{2}ln\left(2\pi \right)\right]-\left[-\frac{1}{2}\left(\sigma^{2}\right)-\frac{{\left(lnTL_{O}-lnTL_{E}\right)}^{2}}{2\sigma^{2}}\right]$$

where *n* is number of data, *i* indicates the number of parameters for each candidate growth model selected (VBG-3, VBG-2, GG-3, and LG-3), *TL_O_* is the total length observed, and *TL_E_* is the total length estimated. For *σ*, the following analytical solution [48] is proposed:

(4) $$\sigma =\sqrt{\frac{1}{n}\overset{n}{\underset{t=1}{\sum }}{\left(lnTL_{O}-lnTL_{E}\right)}^{2}}$$

### 2.9. Confidence Intervals

To estimate the confidence intervals (CI) of the *θ_i_* parameters in the candidate growth models, two approaches were used: (1) the likelihood profile method [48] for the parameter *t*_0_ because there is no correlation between parameters and (2) the likelihood contour method when there is a correlation between parameters [49,50,51], which is observed in the parameters *L*_∞_ and *k*. For the likelihood profile method, a chi-square distribution with 1 degree of freedom (df) was used, and therefore, values equal to or less than 3.84 were accepted within the CI. For the likelihood contour method, a chi-square distribution with 2 df was used, and values equal to or less than 5.99 were accepted within the CI [30]. The CIs were estimated based on Haddon [49].

### 2.10. Model Selection

Model performance was evaluated using Akaike’s Information Criterion (AIC), where the best model was the one with the lowest AIC_c_ value. For model comparisons, the delta *AIC* (Δ*AIC*) and Akaike weights (*w_i_*) were calculated [42]. The Δ*AIC* is a measure of each model relative to the best model and is calculated as Δ*AIC* = *AIC_i_* − min*AIC*, where *AIC_i_* is the *AIC* value for model *i* and min*AIC* is the *AIC* value of the best model. Models with Δ*AIC* of 0–2 had substantial support, while models with Δ*AIC* of 4–7 had considerably less support, and models with Δ*AIC* > 10 had essentially no support. Akaike weights (*w_i_*) represent the probability of choosing the correct model from the set of R-candidate models and was calculated as:

(5) $$w_{i}=\frac{e^{-0.5\Delta AIC}}{\sum_{i=1}^{R}e^{-0.5\Delta AIC}}$$

## 3. Results

### 3.1. Collection of Samples

**Figure 2:**
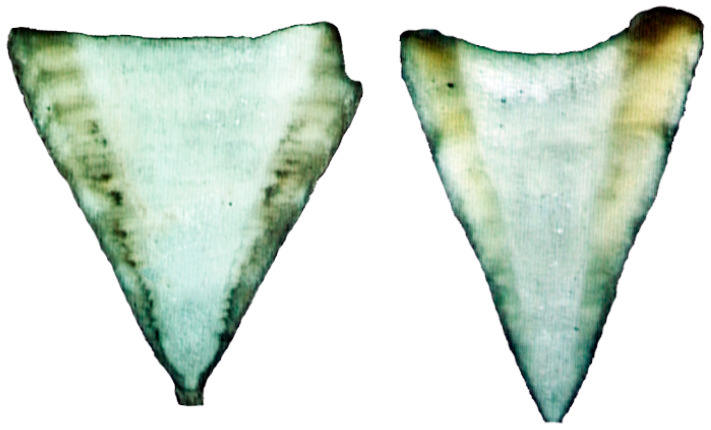
Sagittal sections of centra where growth bands were not evident and could not be read.

A total of 305 specimens (260 females and 45 males) were initially used for the aging study. Of the processed vertebrae, 245 (80%) were readable from 209 females ranging in size from 49 to 84 cm TL and 36 males ranging from 41.5 to 58.8 cm TL, with females being significantly larger than males (D = 0.84, *p* < 0.001) and having a well-represented sample for each month of the year (Table 1). Growth bands were poorly visible in the intermedialia zone (Figure 2); therefore, reading was performed on the corpus calcareum. Significant linear relationships between VR and TL (*p* = 0.001) were found for both sexes (females: TL = 0.08VR−0.65, r^2^ = 0.81; males: TL = 0.07VR−0.2, r^2^ = 0.67), verifying that these vertebrae were suitable structures for age determination. The mean radius of the observed birth band was 0.79 ± 0.08 mm (mean and S.E.). Similarly, the mean VR of the near-term embryo was 0.69 ± 0.02 mm TL, indicating that the birth band was identified correctly.

### 3.2. Precision and Accuracy

**Figure 3:**
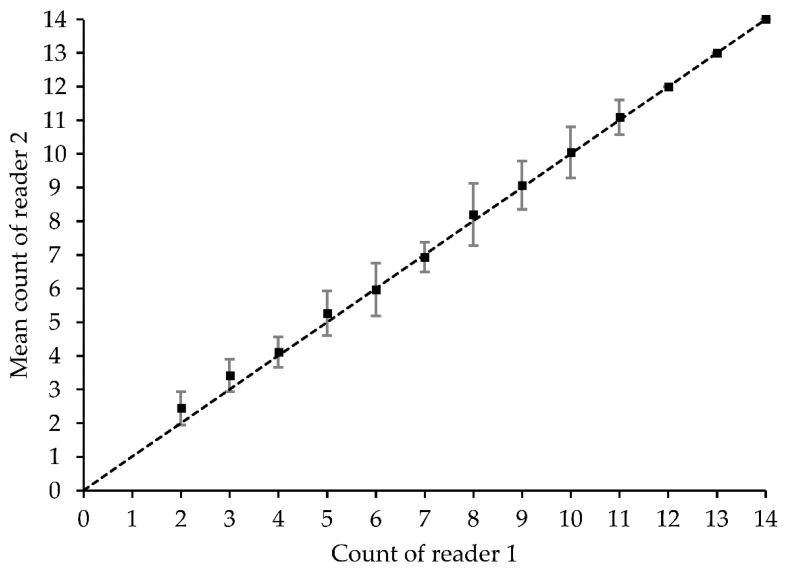
Age-bias plot of reader band-pair counts. Dots with standard deviation bars are the mean counts of reader 2 relative to reader 1. The diagonal line indicates a one-to-one relationship.

Age estimates agreed closely between readers. Age band counts resulted in an APE between readers of 3.3% and CV of 4.7%, with a PA of 69%, PA ± by one band of 95% and PA ± by two bands of 100%. Both age-bias plots (Figure 3) and Bowker’s test of symmetry (X^2^ = 14.5, df = 9, *p* = 0.89) indicated no systematic differences between readers. These precision and accuracy values indicate a high level of reproducibility.

### 3.3. Periodicity of Band Formation

**Figure 4:**
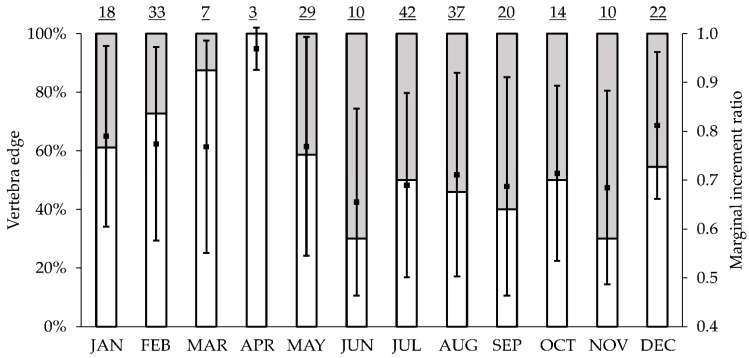
Monthly variations in vertebra edge and marginal increment ratios (MIR). Monthly frequency of translucent (□) and opaque bands (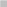) determined from thin sections and monthly variation of mean MIR. Sample size is indicated on top of the graph; (▪) mean monthly MIR; bars show the standard error for monthly values.

**Figure 5:**
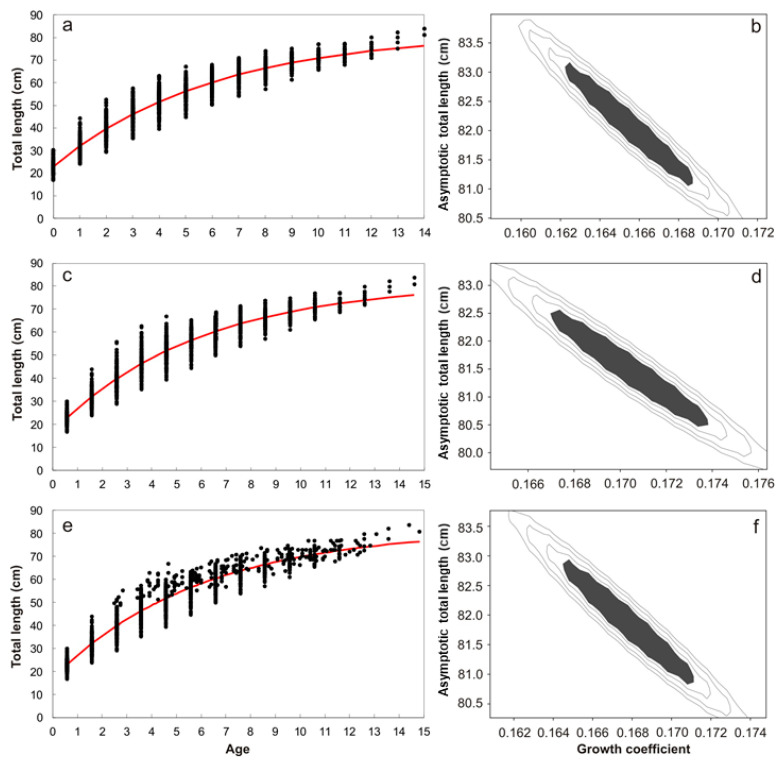
Growth curves and likelihood contours for *L*_∞_ (asymptotic total length) and k (growth coefficient), estimated for VBG-3 for three different data sets: unadjusted (**a**,**b**), adjusted to reproductive cycle (**c**,**d**) and adjusted to reproductive cycle and date of capture (**e**,**f**). For growth curves, black dots represent the observed and back-calculated data, and the red lines represent estimated growth. For likelihood contours, shaded areas denote the confidence region for *L*_∞_ and *k*, assuming a chi-square distribution with n = 2 df.

The categorization of growth bands at the edges of the vertebrae as opaque or translucent was possible. There were no differences in monthly marginal increments (Kruskal–Wallis: H_11, 246_ = 16.7, *p* = 0.11), although a pattern was observed in both marginal increment analysis and edge analysis (Figure 4). The mean MIR and the translucent edge percentage were highest during April and lowest during June, suggesting that a single band pair is formed annually on the vertebral centra of *N. entemedor* during April. Assuming the formation of a pair of bands each year, 14 age groups were identified for *N. entemedor* (Table 2 and Table 3), as well as the formation of a birth band right after birth. The fourth and fifth age groups were predominant, whereas the 14–15 year groups were poorly represented (Figure 5a,c,e). The one-year age group was represented only by males (n = 2), and in the 2-year age group, males were more frequent than females. For the 3–6 year age groups, females were increasingly more frequently than males, which were absent in the remaining age groups. The age structure was different between sexes (D = 0.66, *p* < 0.001).

### 3.4. Growth Estimation and Model Selection

Due to a low sample size of males, growth models were only adjusted to the observed and back calculated age-length data of females. Growth models fitted to the data are shown in Table 4, with CIs for growth parameters *L*_∞_ and k for each one of the data sets analyzed. Their likelihood contours are shown in Figure 5b,d,f. Based on *AIC* values, the VBG-3 presented the best fit to the data to describe the growth of *N. entemedor* females for every data set analyzed (unadjusted and adjusted ages; Table 4 and Figure 5a,c,e). Furthermore, the VBG-3 was the only fitted model with empirical support (Δ*_i_* = 0) for every data set analyzed, while the rest of the models had no support (Δ*_i_* > 10). In each dataset, VBG-3 obtained the maximum −log-likelihood values. The VBG-3 unadjusted-age data set showed the maximum −log-likelihood value among the three, while the rest of the parameters varied slightly between data sets, *L*_∞_ ranged from 81.5 to 82.1 cm TL, and the estimates were close to the observed maximum length (TL = 84.0 cm); k remained constant at 0.17 cm year^−1^.

## 4. Discussion

We found sexual dimorphism by size between sexes, with females (TLmax = 84 cm) being larger than males (TLmax = 58 cm). Similar differences in sizes between sexes were described for *N. entemedor* by Villavicencio-Garayzar [24] off the west coast of Baja California Sur (Bahía Magdalena) and for other closely-related species, such as *N. brasiliensis* [52], *Torpedo californica* [53], *T. marmorata* [54] and *T. nobiliana* [55]. This suggests a selective advantage for larger-sized females; as has been mentioned, the larger size in female elasmobranchs facilitates the accommodation and nourishment of embryos [56]. Similarly, the differences in estimated maximum ages between sexes (6 years for males, 14 years for females) suggests both sexes are not equally long-lived. Furthermore, elasmobranch sexual segregation has been documented related to sex differences in body size, which possibly confers differences in attributes such as predation risk and nutritional requirements [57]. Therefore, the absence of males TL > 59 cm (and >6 years old) in our sample could be explained by differential mortality (or differential longevity) or by sexual segregation. In addition, gear selectivity can be ruled out as a factor for the observed differences because the same gear was used during the full study; thus, differences in size and age by sex seem to be characteristic of the species.

In recent decades, the need to perform age validation has been stressed [31,58]. Villavicencio-Garayzar [24] verified the annual deposition through marginal increments for *N. entemedor* off the west coast of Baja California, concluding that annual band formation occurs during June. Moreover, annual deposition has been verified through marginal increment analysis for another Torpediniformes species, *Torpedo marmorata* [54], as well as for other batoid species such as *Dipturus trachyderma* [59], *Bathyraja parmifera* [60] and *Urotrygon rogersi* [7]. Accounting for this, annual deposition of a pair of bands was assumed in our study; however, we suggest being cautious since MIR did not show monthly significant differences. A pattern was observed both in MIR and edge analyses which suggests that annual band deposition concludes in April.

Although the VBG-3 model in the unadjusted age data set showed the maximum −log-likelihood value among the three data sets, the length at birth described by this model, as estimated from the intersection of the growth curve with the length axis (*L*_0_ = 23.0 cm TL), was the farthest from the length of birth described for the species in the southern Gulf of California (14.5 cm LT; [28]). Considering that the rest of the parameters varied slightly between data sets, according to statistical results (–log-likelihood, *AIC* and *W_i_*) and biological interpretation (*L*_∞_, *k* and *L*_0_ values), we considered the VBG-3 based on the adjusted age to date of capture (age Adjustment 2) to be the growth model that best describes individual growth of *N. entemedor*. The multimodel inference approach allows the analysis of an alternative growth hypothesis for *N. entemedor* and avoids the risk of using an inappropriate model *a priori* [61].

The estimated ages for females in our study, 2–14 years, were similar to ages, 1–15 years, estimated for females by Villavicencio-Garayzar [24] off the west coast of Peninsula Baja California, Mexico. Nonetheless, the ages found for males in our study were 1–6 years (LT = 41–59 cm), which differed from males aged 1–11 years (LT = 24–67 cm) obtained by Villavicencio-Garayzar [24]. This could be due to differences in longevity between study areas related to differences in environmental conditions (e.g., temperature; [62]) or differences in the spatial distribution of older males since sexual segregation among elasmobranchs have been documented related to sex differences in body size [57].

The *L*_∞_ estimated for females in our study (TL = 81.87 cm) was similar to *L*_∞_ = 82.6 cm TL estimated for females by Villavicencio-Garayzar [24] and close to the maximum observed length (TL = 84 cm). On the other hand, the growth coefficient estimated for females in our study, *k* = 0.17 year^−1^, was lower than *k* = 0.30 year^−1^ obtained for females by Villavicencio-Garayzar [24]. In this regard, it has been documented that a limited representation of the sizes in the sample, particularly of small and/or large individuals, can bias parameter estimates using the VBG [63]. However, in our case, the back-calculated lengths at previous ages provided a good representation of younger organisms, and this was observed in the closeness of our *L*_0_ estimation to the observed birth length for *N. entemedor*. Compared with closely-related ray species, our estimation was similar to *k* = 0.18 year^−1^ found for combined sexes of *T. marmorata* [54] but proved higher than *k* = 0.07 year^−1^ obtained for *T. californica* females [53].

## 5. Conclusions

Acknowledging the difficulty of sampling young individuals, we suggest the use of back-calculation estimations where information associated with early stages is limited. Furthermore, we conclude that age adjustment is a useful practice. Although age has traditionally been analyzed on a yearly basis (as a discrete variable), our study indicated that adjusting age to biological features, such as birth date, catch date and the periodicity of growth band deposition, may result in a more precise description of individual growth. Finally, we conclude that *N. entemedor* is a moderate body size elasmobranch species with moderate longevity and fast growth, which is a life history pattern typical of species that grow quickly to overcome mortality in the early life stages.

## Figures and Tables

**Table 1:** Monthly sample size of *Narcine entemedor* specimens analyzed in the present study.

Month	January	February	March	April	May	June	July	August	September	October	November	December
Sample size	18	33	7	3	29	10	42	37	20	14	10	22

**Table 2:** Age–length distribution for *Narcine entemedor* obtained from growth band counts of vertebrae sections. Age estimation follows the age adjustment 2 (adjusted to reproductive cycle and date of capture).

Total Length (cm)	Age Group (Down-Rounded)														Total
	1	2	3	4	5	6	7	8	9	10	11	12	13	14	
42		1													1
44		2													2
46															0
48	2	1	1												4
50		1	3		1										5
52		5		5											10
54		1	4	5		1									11
56		1	1	10	3										15
58			4	6	3	3	1								17
60			3	7	9	3									22
62			1	3	12	6									22
64			1	3	7	2	2	1							16
66					3	5	7	3	1						19
68				1		3	5	1	3	1					14
70						3	5	2	5	6	1				22
72							4	1	6	8	2	1			22
74								3	4	8	5	3			23
76								2	2	1	2	2			9
78										2	4	2			8
80															0
82													1	1	2
84														1	1
Total	2	12	18	40	38	26	24	13	21	26	14	8	1	2	245

**Table 3:** Age–length distribution for *Narcine entemedor* obtained from growth band counts of vertebrae sections and back-calculated. Age estimation follows the age adjustment 2 (adjusted to reproductive cycle and date of capture).

Total Length (cm)	Age Group (Down-Rounded)															
	0	1	2	3	4	5	6	7	8	9	10	11	12	13	14	Total
16										1						1
18	3															3
20	10															10
22	59															59
24	78															78
26	67	4														71
28	14	19														33
30	9	37	2													48
32	5	48	3													56
34		63	10													73
36		31	25	2												58
38		27	34	5												66
40		6	48	7	1											62
42		7	49	16	2											74
44		2	25	31	5											63
46		1	23	41	8	2										75
48		2	16	44	19	4										85
50			3	38	32	7										80
52			8	22	45	11	4									90
54			1	13	33	24	8									79
56			1	9	40	31	8	3								92
58				6	21	32	25	7	1							92
60				3	13	34	19	12	2							83
62				1	4	23	26	19	6	1						80
64				1	3	14	22	21	18	1						80
66						3	16	23	15	12	1					70
68					1		7	12	21	15	5					61
70							3	11	14	17	14	3				62
72								5	5	16	12	5	1			44
74									3	8	12	7	4			34
76									2	2	4	4	3			15
78											2	5	3	1		11
80																0
82													1	1	1	3
84														1	1	2
Total	245	247	248	239	227	185	138	113	87	73	50	24	12	3	2	1893

**Table 4:** Growth model parameter estimates of female *N. entemedor* using three different data sets (unadjusted, adjusted to reproductive cycle (RC) and adjusted to the reproductive cycle and date of capture (DC); see text for details). *L*_∞_ is the theoretical asymptotic size, *k* is the growth coefficient, *L*_0_ is the birth size estimated at age 0, *t*_0_ is the hypothetical age at length zero, −LL is negative log-likelihood, *AIC* is Akaike’s Information Criterion, Δ*_i_* is the Akaike difference, and *W_i_* is the *AIC* weight estimates. CIs estimated from log-likelihood contours are shown in parentheses.

Dataset	Model	*L*_∞_	*k*	*t*_0_	*L*_0_	−LL	*AIC*	Δ*_i_*	*W_i_*
Unadjusted	VBG-3	82.09 (80.59–83.69)	0.165 (0.170–0.173)	−1.988 (−2.008–−1.968)	23.01	3507.23	−7008.45	0.00	1.00
	VBG-2	69.35 (67.25–71.65)	0.314 (0.294–0.334)	-	14.50	2158.51	−4313.03	2695.43	0.00
	GG-3	73.49 (72.89–74.14)	0.301 (0.292–0.310)	0.46 (0.437–0.477)	23.32	3486.66	−6967.31	41.14	0.00
	LG-3	69.79 (69.44–70.14)	0.444 (0.435–0.453)	1.500 (1.475–1.525)	23.69	3439.89	−6873.77	134.68	0.00
Adjusted-RC	VBG-3	81.50 (80.0–83.10)	0.170 (0.165–0.175)	−1.363 (−1.383–−1.343)	16.89	3413.10	−6820.20	0.00	1.00
	VBG-2	75.52 (74.02–77.12)	0.215 (0.206–0.224)	-	14.50	3352.04	−6700.08	120.11	0.00
	GG-3	73.16 (72.51–73.81)	0.308 (0.299–0.317)	1.020 (0.999–1.039)	18.60	3395.95	−6785.90	34.30	0.00
	LG-3	69.53 (69.39–70.19)	0.454 (0.434–0.455)	2.040 (2.017–2.067)	19.71	3354.06	−6702.12	118.08	0.00
Adjusted-DC	VBG-3	81.87 (80.37–83.47)	0.168 (0.163–0.173)	−1.384 (−1.404–−1.364)	16.96	3454.00	−6902.00	0.00	1.00
	VBG-2	75.59 (74.09–77.19)	0.213 (0.204–0.222)	-	14.50	3386.07	−6768.15	133.85	0.00
	GG-3	73.36 (72.71–74.01)	0.305 (0.296–0.314)	1.030 (1.011–1.051)	18.65	3436.19	−6866.39	35.61	0.00
	LG-3	69.70 (69.35–70.05)	0.449 (0.439–0.460)	2.063 (2.040–2.090)	19.77	3392.80	−6779.60	122.40	0.00

## Data Availability

Not applicable.
